# Determinants of local government deficit: evidence from Spanish municipalities

**DOI:** 10.1016/j.heliyon.2022.e12393

**Published:** 2022-12-17

**Authors:** Javier Cifuentes-Faura, Mihaela Simionescu, Beata Gavurova

**Affiliations:** a University of Murcia, Faculty of Economics and Business, Spain; b Institute for Economic Forecasting of the Romanian Academy, Bucharest, Romania; c Technical University of Košice, Faculty of Mining, Ecology, Process Control and Geotechnologies, Slovakia

**Keywords:** Deficit, Municipalities, Determinants, Budget, Local governments

## Abstract

Understanding the determinants of fiscal deficits is justified by the fact that persistent deficits rapidly lead to the accumulation of public debt. Therefore, the aim of this paper is to analyze the factors that explained the fiscal deficits of Spanish municipalities in the period 2011–2020. The deficit at the municipal level for Spain is explained by considering several determinants covering socioeconomic and political dimensions, such as GDP per capita, unemployment rate, population, political participation, political sign of the ruling party or political force, among others. The method of moments quantile regression (MMQ) and mean group (MG) estimator are applied for the overall sample and for each group of municipalities. In addition, the causality between the deficit and the explanatory variables is analyzed using the Juodis et al. (2021) test. It is found that economic growth only has a long-term beneficial effect on the deficit as it reduces the deficit at all quantile levels except at the 10% quantile. Unemployment increases the deficit in both the short and long run. Political participation and right-wing political parties contribute to the growth of the deficit in the higher quantiles. To reduce the budget deficit, the analysis shows that unemployment should be reduced and economic growth should be boosted. The results are robust to those based on mean group estimators. With this paper, we contribute to the scarce literature on deficit determinants by analyzing the determinants for Spanish municipalities. Furthermore, our findings have important implications for politicians, citizens and stakeholders.

## 1 Introduction

The maintenance of the welfare state is conditional on the exercise of the public function being fundable. The government must establish a balance between the amount allocated to the various items of expenditure to meet the needs of citizens and revenues, mainly from taxes and social security contributions. If the savings of public administrations are insufficient to finance public investment, the resulting budgetary imbalance gives rise to a public deficit (Espinola, 2009).

From the 1980s onwards, continued deficits in the finance of Spain’s public sector’s public sector have been observed, leading to increases in public debt. This situation is a concern for both politicians and research.

In the last decade, Spanish local councils have had to control huge budget deficits arising from the difficulty of controlling spending on services such as health, education or dependency, etc. At the same time, they have had to face financial and funding problems in order to make some of the social services entrusted to them sustainable.

Fiscal decentralization is a crucial determinant of the share of the public budget devoted to capital (Alegre, 2010). Spain is a highly decentralised country with a distribution of revenues and expenditures among the different levels of government that resembles federal states (Germany, Austria, Belgium, etc.), which have three levels of government: central, autonomous and local. The Spanish case is interesting from a global perspective as a consequence of the decentralization process carried out in recent decades, which involved a significant degree of fiscal devolution to heterogeneous regions and the adoption of different national and supranational fiscal rules (Delgado-Téllez and Pérez, 2020). In addition, Spanish local governments have traditionally been very active in capital markets and have accumulated significant levels of public deficits during the economic crisis (Delgado-Téllez and Pérez, 2020).

The Spanish system demonstrates that a deep decentralization process can be achieved in a short time and shows that a decentralized system can be as egalitarian as a centralized one if differences in fiscal capacities and spending needs are corrected (Lago-Peñas and Fernández-Leiceaga, 2014; Lago-Peñas et al., 2017).

Current Spanish legislation obliges local authorities to be accountable and to maintain a balanced budget. It also obliges them to carry out different stages of budgetary planning characterised by the implementation of budgetary measures, structural reforms and the submission to rigorous monitoring conditions.

However, the control of deficits and debt remains a complex problem despite the fact that, through the analysis of the different revenue and expenditure budget items, it is possible to reliably ascertain budgetary stability, the origin of revenue and the sources of financing (Ferraresi et al., 2018; Alexandre et al., 2021).

Excessive fiscal deficits are one of the root causes of the debt crisis. The management and control of public deficits has a huge impact on the financial, economic and political integration of a country, so those governments with excessive public deficits must establish fiscal rules to ensure the sustainability of budgets and public finances (Maltritz and Wüste, 2015). Given the importance of public deficit control and sustainable finances, it is important to determine and know what factors or determinants can affect the level of public deficits in a country.

There are quite a few works on budget deficits, but very few that analyze the determinants of the deficit. Moreover, there is even less work at the local level due to the difficulty of collecting data. With this paper, we contribute to the scarce literature on deficit determinants by analyzing the determinants of deficit for Spanish municipalities, thus filling this gap in the literature. Furthermore, our findings have important implications for politicians, citizens and stakeholders.

Specifically, this paper focuses on analysing the variables that can explain the fiscal deficits of Spanish local councils. It is important to know and analyse these variables, since continued deficits lead to increases in public debt, which can compromise the capacity of public budgets to act as a countercyclical mechanism to stabilise the economy (Correia et al., 2008). The variables analysed in an attempt to explain the deficit cover political, economic and social dimensions. This paper offers an empirical analysis, in the period 2011–2020 for the Spanish local governments. Taking into account that the size of municipalities may require different policies to manage the deficit problem, the analysis focuses on Method of moments quantile regression (MMQ regression) for all municipalities and then separate models for each group of municipalities according to population.

The study shows that both political and economic factors are important determinants of the fiscal deficit. The paper is structured as follows. Section 2 presents a summary of the literature on the determinants of public deficit. Section 3 develops the methodological aspects underlying the analysis. The empirical results are discussed in Section 4. Finally, the conclusions of the study are summarised in Section 5.

## 2 Literature review

A government deficit is when a government spends more than it takes in over a given period of time. In such cases, the country or region is unable to raise enough money to meet its expenditures and incurs debts. The budget (fiscal) balance equals the revenue minus the expenditure of the consolidated government. A negative value means that the government has a deficit, while a positive value shows a surplus. Often the value is expressed as percent of GDP to relate it to the size of the economy.

There are quite a few papers on budget deficits, but most of this literature focuses more on the consequences of deficits on certain macroeconomic variables than on the determinants (Mawejje and Odhiambo, 2020).

Understanding the determinants of fiscal deficits is important given that persistent deficits quickly lead to the accumulation of public debt, especially in low- and middle-income countries (World Bank-IMF, 2018). Public deficits also influence the level of a country’s public debt (Bittencourt, 2015; Gargouri and Ksantini, 2016; Delgado-Téllez and Pérez, 2020; Ioannou, 2022; Nagou et al., 2021). In order to cover its deficit, the government needs to accumulate public debt, having to borrow money from third parties. Given the persistent increase in fiscal deficits in many countries, an important challenge is to control the volatility of the fiscal deficit, and it is therefore advisable to understand the factors that lead to higher volatility.

Several theories have been postulated for the study of the determinants of debt. Ricardian theory considers that fiscal deficits are not determined by any macroeconomic factor and have no long-term macroeconomic consequences (Seater 1993; Tung, 2018; Ahmed and Alamdar, 2018; Nwanna and Umeh, 2019; Ssebulime and Edward, 2019; Usman et al., 2020). Keynesian theory establishes a link between fiscal deficits and growth. A quick scan of the results obtained in the literature in this line does not show a clear consensus on its effect. Gupta et al. (2005), Bose et al. (2007) or Taylor et al. (2012) conclude that there is a positive impact of fiscal deficits on economic growth, while other studies such as Brender and Drazen (2008), Roy and Van den Berg (2009) or Fatima et al. (2011) claim a negative impact. However, Dalyop (2010) and Abd Rahman (2012) argue that the fiscal deficit has no impact on economic growth.

The Neoclassical model considers that the market determines both borrowing and lending decisions, which explains the budget deficit (Diamond, 1965). It also links the budget deficit to the current account deficit. The twin deficit hypothesis states in its form most popularised by the “New Cambridge School” that the former causes the latter (Bird et al., 2019). The twin divergence hypothesis posits that budget deficits arise from current account deficits (Kim and Roubini 2008), Another view states that different patterns of budget deficits are explained by differences in political economy. For example, citizens may value deficit-financed public spending positively, but underestimate its costs in terms of future tax burdens (Nordhaus, 1975). It may also be the case that a politician, faced with the risk of not being re-elected in an upcoming election, may run a higher deficit to finance higher spending.

In recent years, natural disasters have been linked to the fiscal position of governments, since when they occur they tend to reduce government revenues and increase public spending (Benson and Clay, 2004). The long-term sustainability of government finances may be affected depending on whether the resulting imbalance is financed by debt or by replacing planned public investment.

Table 1 shows a summary of the literature review of the determinants of the deficit in recent years. The conclusions derived from these studies do not always agree. Many of these differences are due to the different spatial scope analysed (the determinants of budget deficits are region-specific) and/or the use of different methodologies and models. Some papers identify the determinants of the size of the deficit by focusing on a particular country (Murwirapachena et al., 2013; Bangura et al., 2016), while others perform the analysis for a group of countries (Combes and Saadi-Sedik, 2006; Tujula and Wolswijk, 2007; Maltritz and Wüste, 2015). Very few are limited to the local or regional level (Artés and Jurado, 2018). Panel data are usually used, and the most commonly applied methodology is the Vector Error Correction Model (Galli and Padovano, 2002; Murwirapachena et al., 2013; Alamet al., 2020) and the Generalized Method of Moments (Combes and Saadi-Sedik, 2006; Yahaya et al., 2021).

**Table 1:** Summary of the literature review on the determinants of public deficit over the last 20 years.

Study	Period	Region	Method	Dependent variable	Main Independent variable
Woo (2003)	1970–1990	57 developed and developing countries	Panel data regression	Public sector surplus (as percentage of GDP)	• GDP growth (+) • Financial depth (−) • Inflation (- in developing country) • Population of over 65 years old (- in developing countries) • Trade openness (/) • Party coalition (ns)
Combes and Saadi-Sedik (2006)	1974–1998	66 developing countries	Generalized Method of Moments (GMM)	Budget surplus	• GDP (/) • Inflation (/) • Trade openness (/) • Trade policy (/) • Trade instability (ns) • Urban population (/)
Tujula and Wolswijk (2007)	1970–2002	22 OECD countries	Panel regressions	Variation Deficit to GDP ratio	• GDP growth (+) • Election year (−) • interest rate (/) • Stock price (/)
Agnello and Sousa (2009)	1980–2006	125 countries	Generalized Method of Moments (GMM)	Standard deviations of the budget deficit (as percentage GDP)	• Population (−) • Inflation (+) • GDP (ns) • Openness (+) • Polity Scale (−) • Political instability (+) • Cabinet Changes (+) • Government Crises (+)
Bayar and Smeets (2009)	1971–2006	European Union	OLS estimation	Deficit-to-GDP ratio	• Unemployment (+) • GDP growth rate (−) • Debt (ns) • Ideology (- if left-wing governments) • Political fragmentation (ns) • Stability of the government (−)
Lis and Nickel (2010)	1985–2007	138 countries	Two-stage least squares model with fixed effects	Change in the budget balance (in terms of GDP)	• GDP growth (+) • Inflation (+) in all country and Developing; (ns) in OECD and EU • Change interest rate (/) • Number of large-scale extreme weather disasters (−) in all country and Developing; (ns) in OECD and EU • Dummy variable taking the value one if at least one extreme weather even (−) in all country and Developing; (ns) in OECD and EU • Election dummy (ns) in all country and Developing; (−) in OECD and EU
Javid et al. (2011)	1984–2010	9 South Asian countries	Generalized Method of Moments (GMM)	Logarithm of volatility of Budget Deficit	• GDP per capita (+) • Deficit to GDP (+) • Inflation (+) • Openness (+) • Political Stability (−)
De Haan et al. (2013)	1984–2003	European Union countries except Luxemburg	Panel fixed effects	Budget balance	• Government fragmentation (/) • Effective number of parties (−) • Economic growth (ns) • Debt (as percentage GDP) (+) • Inflation (ns) • Stability and Growth Pact (+) • Institutional quality (+)
Murwirapachena et al. (2013)	1980–2010	South Africa	Vector Error Correction Model (VECM)	Budget deficit (as a percentage of GDP)	• Unemployment (-) • Foreign debt (+) • Investment (−) • Foreign exchange reserves (−) • GDP (+)
Maltritz and Wüstev (2015)	1991–2011	27 EU Countries	Panel data regression	Primary budget balance (difference between government’s revenues and expenditures excluding interest payments for outstanding debt)	• GDP growth (+) • Unemployment (−) • Share of population over 65 (−) • Election dummy (−) • Ideology (ns) • Fiscal Rule Index (+) • Fiscal Council Index (ns) • Stock-flow adjustments (+) • Debt (+) • Inflation (−)
Bangura et al. (2016)	1980–2014	Sierra Leone	Vector error correction model (VECM)	Budget deficit	• GDP (+) • nflation (−) • Interest rate (+) • Investment (−) • Exchange rate (+)
Epaphra (2017)	1966–2015	Tanzania	Vector autoregression (VAR) –Vector error correction model (VECM)	Budget deficit	• GDP (−) • Exchange rate (−) • Inflation (+) • Money supply (+) • Lending interest rate (+)
Alejos (2018)	2000–2015	168 countries	Fixed-effects panel data	Change in the budget deficit (as a percentage of GDP)	• Change in the debt-to-GDP ratio (+) • GDP growth (+) • Inflation (+) • Election year (ns) • Disaster dummy (−)
Artés and Jurado (2018)	2003–2010	3147 Spanish municipalities	Panel regression discontinuity	Primary budget surplus (as a percentage of total revenues)	• Majority (+) • Population (ns) • Density (ns) • Ideology (ns)
Alam et al. (2020)	1980–2018	Bangladesh	Vector Error Correction Model (VECM)	Budget deficit	• Exchange rate (+), Inflation (+), volume of trade (+), money supply (+), GDP (−) in long-run • GDP (−), inflation (−) and money supply (−) in short-run
Gnimassoun and Do Santos (2020)	1998–2017	110 emerging and developing countries	Extreme Bound Analysis (EBA) method and Bayesian Model Averaging (BMA)	Fiscal balance (as a percentage of GDP)	• External shocks (+) • Debt ratio (−) • financial development (−) • Level of democracy (−) • Government control over expenditures (+)
Shehu and Adamu (2021)	1981–2016	Nigeria	Vector Error Correction model (VECM)	Budget deficit (as a percentage of GDP)	• Exchange rate (+) • Interest rate (ns) • External debt (ns)
Yahaya et al. (2021)	2008–2018	Economic Community of West African States (ECOWAS)	Two-step dynamic GMM	Budget deficit	• Debt to GDP ratio (+) • Inflation (+) • Rainfall (+) • Temperature (ns) • GDP growth (−)

(ns): not significant; (+): positive correlation; (−) negative correlation; (/) the sign and significance depends on the specific model and/or country analyze.

In line with the different theories discussed above, in the review of the empirical literature on the determinants of fiscal deficits we find macroeconomic, political, institutional, structural and environmental factors. Macroeconomic variables include interest rates (Laubach, 2009); inflation (Agnello and Sousa, 2009); exchange rates (Kim and Roubini, 2008), unemployment (Murwirapachena et al., 2013) and economic growth (Adam and Bevan, 2005). The quality of economic management and budgetary institutions have also been considered in determining the size of deficits (De Haan et al., 2013).

Lis and Nickel (2010) show that the lagged debt ratio, GDP growth and inflation are statistically significant and positively related to budget balances. Woo (2003) concludes that inflation has a negative influence in developing countries and is not significant in developed countries. In Combes and Saadi-Sedik (2006) the association between inflation and budget surplus is not clear depending on the estimated model. Maltritz and Wüste (2015) conclude that higher borrowing improves the budget balance and reduces deficits. They also find that there is no significant effect of GDP growth and that there is a higher deficit when unemployment rates are higher.

Among the political factors, deficits have been related to political motivations, political strength or political ideology. Mink and De Haan (2006) find that budget deficits increase in election years, whereas they do not in the previous year. Andrikopoulos et al. (2004) show that right-wing governments focus on fiscal stabilisation. Agnello and Sousa (2013) argue that political instability is positively associated with higher government deficit volatility. They also find that trade openness and inflation increase the volatility of the budget deficit, especially in small countries. Maltritz and Wüste (2015) find higher deficits during election years, while Lis and Nickel (2010) find a negative relationship only for OECD and EU countries. Ngo and Nguyen (2020) find that fiscal deficits are higher in countries with frequent changes of government. Artés and Jurado (2018) find that single-party majorities run budgets with significantly larger primary surpluses than in the presence of coalitions, and that lower deficits are mainly due to the ability of majority governments to raise more revenue.

Structural elements such as population have also been examined. Woo (2003) concludes that in developing countries a larger population of over 65 years old has a higher deficit, although in developed countries this relationship is not significant. Maltritz and Wüste (2015) also find a positive relationship between the level of deficit and the percentage of population over 65 years old. In Combes and Saadi-Sedik (2006) the degree of urbanisation has an indeterminate effect on the fiscal surplus.

Finally, climate change and the impact of extreme weather conditions on the public deficit have also been studied (Lis and Nickel, 2010; Alejos, 2018; Bachner et al., 2019; Yahaya et al., 2021). Lis and Nickel (2010) show that there are differences in the response of fiscal deficits to weather shocks. And one of the main implications of the work of Yahaya et al. (2021) is that extreme and unpredictable precipitation will lead to an increase in the budget deficit.

Our work focuses on analyzing the variables that can explain the fiscal deficits of Spanish municipalities, distinguishing according to the size of the municipality (number of inhabitants), to the best of our knowledge, there is no work in this line for Spanish municipalities. In this way, the literature in this field is enriched, with the aim of enabling policy makers to understand the determinants of public deficits and to propose solutions to control the increase in public deficits.

## 3 Methodology and data

The budget deficit characterizes the situation when the public expenditure is higher than revenues. The deficit at municipality level in the period 2011–2020 for Spain is explained by considering various determinants that cover political, economic and social dimensions. The consideration of different explanatory variables is conditioned by the data availability for Spanish municipalities: political participation (number of voters divided by the electoral census), type of political party (right wing (1) and left wing (0)), political majority (it takes the value 1 in case of majority for party that is at governance in that municipality and 0 if the majority is not reached), Herfindalh index as a measure of political strength (sum squared of the percentage share of votes associated to any party; this index ranging between 0 and 1 shows strong government for values near the upper limit of the interval), GDP per capita considered as a measure for standard of living and economic prosperity, population more than 65 years old, population under 15 years old, unemployment rate.

Since the size of municipalities might require different policies to manage the deficit issue, the analysis focuses on quintile regressions for all municipalities and then separate models for each group of municipalities according to population number. Moreover, Balaguer-Coll et al. (2016) showed that the determinants of debt or deficit might be different in large municipalities compared to small ones.

The Spanish clusters or groups of municipalities are denoted as group 1 (<5000 people), group 2 (between 5000 and 20,000 inhabitants), group 3 (between 20,000 and 50,000 people) and group 4 (more than 50,000 inhabitants). All the data series are transformed by applying the natural logarithm, excepting dummy variables like political majority and political party. The rate of growth is approximated by the logarithm of the indicator that allows us to make interpretations in terms of elasticities in the panel data models. Descriptive statistics are provided for deficit growth in the overall sample and in each group of municipalities (Table 2). Each cluster counts more than 5800 municipalities and the highest average deficit growth is registered by the group with the most populated municipalities. The maximum increase in deficit was also observed in this group of units (almost 9.2). Stata 15 software was used to make all the computations.

**Table 2:** Descriptive statistics for natural logarithm of deficit.

Group	Number of municipalities	Mean	Standard deviation	Minimum	Maximum
Overall group	23,312	3.17	3.88	-7.73	9.19
Group 1	5833	2.91	3.70	-6.80	6.88
Group 2	5831	4.01	4.31	-7.06	7.56
Group 3	5815	4.64	4.46	-7.58	7.45
Group 4	5833	5.19	4.73	-7.73	9.19

If the data series do not follow a normal distribution, panel quantile regressions are recommended to explain deficit in Spanish municipalities. All the variables in the models are considered in the logarithmic form, excepting political party (denoted by Party) and political majority (Majority). These variables are: deficit (Deficit), GDP per capita (*GDP*), unemployment rate (*Unemployment*), political participation (*Participation*), Herfindalh index (*Herfindalh*), population more than 65 years old (*Pop65*), population under 15 years old (*Pop15*). The potential multicollinearity was checked by computing the matrix of correlation between the variables in log form and the variance inflation factors as other studies also suggest (Abonazel and Shalaby, 2020, 2021). The results are presented in Appendix 1 and indicate no multicollinearity.

(1) $${Deficit}_{it}=f\left({GDP}_{it},{Unemployment}_{it},{Participation}_{it},{{Herfindalh}_{it},Majority}_{it},{Party}_{it},{Pop15}_{it},{Pop65}_{it}\right)$$

t index for time (t = 2011, 2012,…, 2020)
i-index for municipality

Starting from the model in (1), panel quantile regressions are built due to the advantages of this approach: no assumptions on the data distribution and on the existence of moment function, presence of unobserved heterogeneity in distribution and cross-sections, improvement in robustness in the presence of outliers or heavy tailed distributions (Salman et al., 2019).

If $\alpha_{i}$ represents the unobserved municipality effects, Eq. (2) is written as:

(2) $${E[deficit}_{it}|\left({GDP}_{it},{Unemployment}_{it},{Participation}_{it},{{Herfindalh}_{it},Majority}_{it},{Party}_{it},{Pop15}_{it},{Pop65}_{it}\right),\alpha_{i}]=\left({GDP}_{it}^{T},{Unemployment}_{it}^{T},{Participation}_{it}^{T},{Herfindald}_{it}^{T},{Mayority}_{it}^{T},{Party}_{it}^{T},{Pop15}_{it}^{T},{Pop65}_{it}^{T}\right)\beta +\alpha_{i}$$

In this case, $\hat{\beta }\left(\tau \right)$ is the $\tau^{th}$ quantile level and the following values are considered for the parameter size τ: 0.10, 0.25, 0.50, 0.75, and 0.95, under different forms of conditional quantile functions as shown in Eq. (3):

(3) $$\hat{\beta }\left(\tau \right)={argmin}_{\beta \in R^{k}}\left[\sum_{i\in \left\{i:y_{i}\geq x_{i}\beta \right\}}\tau \left|y_{i}-x_{i}\beta \right|+\sum_{i\in \left\{i:y_{i}<x_{i}\beta \right\}}\left(1-\tau \right)\left|y_{i}-x_{i}\beta \right|\right]$$

τ takes values in the interval [0; 1] and indicates the least value of the weighted sum of deviation in absolute value. As shown in Eq. (4), the conditional quantile of the deficit for the explanatory variables $x_{i}$ is:

(4) $$Q_{EP}\left(\tau |{GDP}_{it},{Unemployment}_{it},{Participation}_{it},{{Herfindalh}_{it},Majority}_{it},{Party}_{it},{Pop15}_{it},{Pop65}_{it}\right)=\left({GDP}_{it},{Unemployment}_{it},{Participation}_{it},{{Herfindalh}_{it},Majority}_{it},{Party}_{it},{Pop15}_{it},{Pop65}_{it}\right)\beta_{\tau }$$

Method of moments quantile regression (MMQ regression) is applied for overall sample and for each group of municipalities to check for robustness. Moreover, the causality between deficit and explanatory variables is analyzed using the Juodis et al. (2021) test. In the case of this test, the Half Panel Jackknife method is applied to remove the Nickell bias corresponding to pooled estimator. Let us start from a linear dynamic panel model to explain ${Deficit}_{i,t}$ based on explanatory variables $x_{i,t}$:

(5) $${Deficit}_{i,t}=\varphi_{0,i}+\sum_{p=1}^{P}\varphi_{p,i}{Deficit}_{i,t-p}+\sum_{p=1}^{P}\beta_{p,i}x_{i,t-p}+\varepsilon_{i,t}$$

$\varphi_{0,i}$- municipality-specific effects
$\beta_{p,i}$- heterogeneous feedback parameters
$\varphi_{p,i}$- heterogeneous autoregressive parameters
$\varepsilon_{i,t}$- error term
i = 1,2,…,N (municipality index); t = 1,2,…,T (time index); p = 1,2,...,P (lag index)

In the case of Eq. (5), some assumptions should be fulfilled: the data should be stationary for all the variables in the model, while cross-sectional dependence could be neglected.

The null hypothesis states that $x_{i,t}$ does not Granger cause ${Deficit}_{i,t}$ ($H_{0}:\;\beta_{p,i}=0$ , for all *i* and *p*), while the alternative hypothesis supposes $\beta_{p,i}\neq 0$ , for some *i* and *p* ($H_{1}$).

## 4 Results

### 4.1 Preliminary tests

The cross-sectional dependence is checked for panel data and in this case it might be due to common specific characteristics of Spanish municipalities related to legislation, types of expenditure etc. The Pesaran CD test is employed to show the existence of the cross-sectional dependence from statistical perspective. The results in Table 3 based on this test show the rejection of cross-sectional independence at 1% significance level for all data series in the overall sample and in all groups of municipalities.

**Table 3:** The results of cross-sectional dependence test.

Variable in natural logarithm	Overall sample	Group 1	Group 2	Group 3	Group 4
Deficit	42.56∗	38.02∗	32.18∗	34.56∗	36.29∗
GDP	30.43∗	29.44∗	25.83∗	26.02∗	28.22∗
Unemployment	17.93∗	14.56∗	17.39∗	16.56∗	15.55∗
Participation	23.65∗	21.85∗	24.81∗	23.44∗	22.45∗
Herfindalh	16.07∗	15.22∗	12.37∗	13.69∗	14.05∗
Pop15	12.06∗	9.77∗	10.74∗	10.60∗	11.34∗
Pop65	17.05∗	9.045∗	13.63∗	16.34∗)	14.46∗

Under cross-sectional dependence, in this unbalanced panel, the presence of unit root is checked using the covariate-augmented Dickey Fuller test (CADF). Table 4 shows that the test is applied for data in level and for data in the first difference.

**Table 4:** The results of CADF unit root test.

Variable in logarithm		Data in level					Data in the first difference			
	Overall sample	Group 1	Group 2	Group 3	Group 4	Overall sample	Group 1	Group 2	Group 3	Group 4
Deficit	-1.32	-1.02	-0.99	-0.87	-1.23	-3.97∗	-3.21∗	-3.99∗	-4.00∗	-4.05∗
GDP	-0.99	-1.13	-0.89	-0.83	-1.30	-4.03∗	-3.34∗	-3.17∗	-4.17∗	-4.39∗
Unemployment	-1.03	-1.71	-1.00	-1.72	-0.99	-4.12∗	-4.12∗	-3.22∗	-4.34∗	-4.01∗
Participation	-1.83	-1.87	-0.93	-1.42	-0.99	-4.56∗	-3.99∗	-3.72∗	-4.67∗	-3.93∗
Herfindalh	-1.23	-1.13	-0.79	-0.93	-1.04	-4.00∗	-4.15∗	-3.12∗	-4.12∗	-3.15∗
Pop15	-1.29	-1.78	-1.33	-0.73	-0.94	-4.20∗	-4.11∗	-3.44∗	-4.18∗	-4.01∗
Pop65	-1.12	-1.06	-1.66	-1.16	-1.02	-3.99∗	-3.89∗	-3.67∗	-4.28∗	-3.53∗

According to Table 4 the data series for all variables present only one unit root at 1% significance level. In other words, the data series are integrated of order 1 and cointegration should be analyzed based on Westerlund test. According to the values of statistics, the cointegration relationship between deficit and explanatory variables is rejected at 10% significance level (Gt = −2.233, Ga = −3.066, Pt = −2.334, Pa = −5.889 (p-values are higher than 0.1)).

If the hypothesis of normal distribution of data is rejected at 5% significance level, MMQ regressions are constructed.

The Shapiro-Wilk and Shapiro-Francia tests are used to check for normal distribution. Table 5 suggests the rejection of normal distribution for overall sample and for each cluster of municipalities at 1% significance level.

**Table 5:** The results of tests for normal distribution (Shapiro-Wilk and Shapiro-Francia tests).

Variable in logarithm		Shapiro-Wilk test					Shapiro-Francia test			
	Overall sample	Group 1	Group 2	Group 3	Group 4	Overall sample	Group 1	Group 2	Group 3	Group 4
Deficit	24.32∗	24.10∗	19.81∗	16.61∗	15.04∗	26.70∗	26.11∗	19.60∗	15.83∗	14.15∗
GDP	16.31∗	15.83∗	11.22∗	7.90∗	6.23 ∗	18.23∗	17.44∗	11.20∗	7.49∗	5.97∗
Participation	19.76∗	19.25∗	14.62∗	10.93∗	9.79 ∗	21.89∗	21.05∗	14.43∗	10.36∗	9.17∗
Herfindalh	16.35∗	15.64∗	11.68∗	9.14 ∗	8.32 ∗	18.30∗	17.27∗	11.70∗	8.83∗	7.95∗
Pop15	12.78∗	12.27∗	8.41∗	5.88 ∗	5.24 ∗	14.36∗	13.56∗	8.05∗	4.89∗	4.16∗
Pop65	15.35 ∗	14.61∗	11.14∗	8.26∗	6.78∗	17.23∗	16.18∗	11.12∗	7.88∗	6.47∗
Unemployment	18.28∗	17.77∗	13.21∗	9.56∗	8.38∗	20.28∗	19.44∗	13.14∗	9.20∗	7.92∗

Considering the results of preliminary tests, MMQ regressions are built for overall sample and for each group of municipalities. In the end, the causality in panel is checked on stationary data in the first difference.

### 4.2 Results for overall sample and robustness check for groups of municipalities

The budget balance reflects the expenditure and revenues made by central/local government during a financial exercise. If the difference between expenditure and revenues is calculated and it has a negative value, budget deficit is registered as the expenditure is higher than revenues. Otherwise, the value is positive and it corresponds to budget surplus. The MMQ regression model for all municipalities is presented in Table 6.

**Table 6:** MMQ regression models to explain public deficit in Spanish municipalities (2011–2020).

Variable in logarithm	Quantile levels				
	10%	25%	50%	75%	95%
GDP	0.4358819 (0.352)	0.2269113∗ (0.001)	0.2087884∗ (0.000)	0.2012981∗ (0.000)	0.1910127∗ (0.000)
Unemployment	-0.315815∗∗ (0.037)	-0.0862545∗ (0.000)	-0.0663458∗ (0.000)	-0.0581176∗ (0.000)	-0.0468187∗ (0.000)
Participation	-9.102163 (0.000)	-1.603895 (0.000)	-0.9536082∗ (0.000)	-0.6848444∗ (0.000)	-0.3157834∗ (0.000)
Herfindalh	-2.356559 (0.000)	-0.3804432 (0.000)	-0.2090648∗ (0.000)	-0.138234 (0.000)	-0.0409706∗ (0.003)
Majority	0.0021106 (0.992)	-0.017439 (0.577)	-0.0191344 (0.234)	-0.0198352∗ (0.075)	-0.0207974∗∗ (0.035)
Party	-0.1399264 (0.338)	-0.0322727 (0.114)	-0.0229365∗∗ (0.029)	-0.0190778∗ (0.009)	-0.0137792∗∗ (0.032)
Pop15	-0.4840923∗∗ (0.011)	0.0053478 (0.841)	0.0477944∗ (0.000)	0.0653376∗ (0.000)	0.0894276∗ (0.000)
Pop65	2.531439 (0.000)	0.2322105 (0.005)	0.0328101 (0.436)	-0.0496022∗∗∗ (0.09)	-0.1627691∗ (0.000)
Constant	35.13132 (0.081)	8.747417∗ (0.000)	6.459276 (0.000)	5.513586∗ (0.000)	4.214984∗ (0.000)

As expected, economic growth reduces the deficit for all quantile levels excepting 10% quantile. This suggests that growth has only a long-run beneficial effect on deficit. On the other hand, unemployment increases the deficit on the short and long-run. Political participation and political party (right wing) contribute to the deficit growth at 50%, 75% and 95% quantiles. Population with more than 65 years old and political majority increases the deficit at 75% and 95% quantile levels. Population under 15 years old increases the deficit only in the short-run at 10% quantile, but starting with the 50% quantile, in the long-run, this population contributes to the reduction in deficit. As expected, children will become in the long term working force that will integrate on labour market and will reduce deficit through paid taxes.

The overall analysis suggests that unemployment should be reduced at national level and the economic growth should be enhanced to reduce the budget deficit. On the other hand, the quality of the governance should improve and the political program should target the reduction of deficit.

The results change when a separate analysis is conducted for each group of municipalities. The robustness check is made for these subsamples according to municipality size.

Economic growth, political participation, population under 15 years and political party reduce the deficit in small municipalities from group 1 (see Table 7). Political strength contributes to deficit reduction only in the long-run starting with the 0.5 quantile. Political majority decrease deficit only for 95% quantile, while population of more than 65 years makes pressure on budget in the long-run with impact in the 75% and 95% quantiles.

**Table 7:** MMQ regression models to explain public deficit in Spanish municipalities from group 1 (2011–2020).

Variable in logarithm	Quantile levels				
	10%	25%	50%	75%	95%
GDP	0.6629814 (0.156)	0.2867286∗ (0.001)	0.2416944∗ (0.000)	0.2159838∗ (0.033)	0.1880553∗ (0.000)
Unemployment	-0.1140569 (0.476)	-0.0114631 (0.697)	0.0008166 (0.957)	0.0078271 (0.421)	0.0154424 (0.173)
Participation	1.215577∗∗∗ (0.098)	0.5208691∗ (0.000)	0.4377185∗ (0.000)	0.3902469∗ (0.000)	0.3386802∗ (0.000)
Herfindalh	-0.0586756 (0.853)	0.0495737 (0.394)	0.0625302∗∗ (0.038)	0.0699273∗ (0.000)	0.0779624∗ (0.001)
Majority	-0.2008845 (0.366)	-0.0159676 (0.696)	0.0061654 (0.771)	0.0188014 (0.165)	0.0325274∗∗ (0.039)
Party	0.2601607∗∗∗ (0.078)	0.0516857∗∗ (0.057)	0.026733∗∗∗ (0.058)	0.0124873 (0.164)	-0.0029874 (0.775)
Pop15	0.4108318∗∗∗ (0.054)	0.128525∗ (0.001)	0.0947353∗ (0.000)	0.0754444∗ (0.000)	0.0544893∗ (0.000)
Pop65	0.8801202 (0.124)	0.0568165 (0.589)	-0.041726 (0.444)	-0.0979849∗ (0.005)	-0.1590971∗ (0.000)
Constant	-9.958278∗∗ (0.018)	0.6329722 (0.414)	1.900656∗ (0.000)	2.624389∗ (0.000)	3.410556∗ (0.000)

Contrary to expectations, the population aged over 65 years reduces the deficit for all quantiles excepting 0.95 in the group 2, which might be explained by the fact that the number of these people tend to reduce in time (see Table 8). Political strength seems to increase deficit at 75% quantile.

**Table 8:** MMQ regression models to explain deficit in Spanish municipalities from group 2 (2011–2020).

Variable in logarithm	Quantile levels				
	10%	25%	50%	75%	95%
GDP	1.401383 (0.396)	0.1857161 (0.235)	0.1246433 (0.149)	0.0911153∗∗∗ (0.096)	0.0496118 (0.292)
Unemployment	0.0558786 (0.922)	0.013544 (0.802)	0.0114172 (0.703)	0.0102496 (0.589)	0.0088043 (0.589)
Participation	1.153455 (0.650)	0.078475 (0.744)	0.02447 (0.854)	-0.0051778 (0.951)	-0.0418781 (0.563)
Herfindalh	-0.7047126 (0.536)	-0.1158482 (0.283)	-0.0862648 (0.148)	-0.0700239∗∗∗ (0.064)	-0.0499198 (0.124)
Majority	0.1777711 (0.818)	0.0269486 (0.713)	0.0193715 (0.632)	0.0152119 (0.553)	0.01 (0.648)
Party	-0.2334066 (0.618)	-0.0287945 (0.555)	-0.0185152 (0.492)	-0.012872 (0.451)	-0.0058864 (0.689)
Pop15	-0.3691499 (0.707)	-0.0478829 (0.494)	-0.031743 (0.412)	-0.0228825 (0.351)	-0.0119143 (0.572)
Pop65	3.757181∗∗∗ (0.074)	0.4265106∗∗ (0.032)	0.259184∗∗ (0.019)	0.1673245∗∗ (0.017)	0.0536138 (0.372)
constant	-18.71732 (0.202)	3.98991∗ (0.004)	5.130678∗ (0.000)	5.756941∗ (0.000)	6.532177∗ (0.000)

In the group 3, unemployment and political participation increase deficit for 25% and 50% quantiles, while populated aged over 65 years enhances budget disequilibrium in the long-run for the last two quantiles (see Table 9).

**Table 9:** MMQ regression models to explain public deficit in Spanish municipalities from group 3 (2011–2020).

Variable in logarithm	Quantile levels				
	10%	25%	50%	75%	95%
GDP	4.219611 (0.262)	0.351089 (0.18)	0.2082384 (0.142)	0.1249678 (0.154)	0.0217452 (0.809)
Unemployment	1.945228 (0.106)	-0.1502403∗∗∗ (0.073)	-0.0839578∗∗∗ (0.067)	-0.0453205 (0.106)	-0.0025747 (0.929)
Participation	-4.35581 (0.418)	-0.5081388 (0.173)	-0.3660582∗∗∗ (0.071)	-0.2832365∗∗ (0.024)	-0.1805702 (0.16)
Herfindalh	0.6212583 (0.776)	-0.0079244 (0.958)	-0.0311579 (0.705)	-0.0447011 (0.378)	-0.0614894 (0.237)
Majority	-0.7513449 (0.644)	-0.0427458 (0.705)	-0.0165797 (0.787)	-0.001327 (0.972)	0.0175803 (0.651)
Party	-0.6232865 (0.575)	-0.0674446 (0.382)	-0.0469194 (0.263)	-0.0349548 (0.176)	-0.0201235 (0.448)
Pop15	-0.5043803 (0.719)	-0.0000748 (0.999)	0.0185474 (0.725)	0.0294026 (0.367)	0.0428589 (0.200)
Pop65	-0.6493312 (0.875)	-0.1232727 (0.668)	-0.1518022 (0.331)	-0.1684326∗∗∗ (0.080)	-0.1890478∗∗∗ (0.056)
Constant	3.972045 (0.900)	7.472826∗ (0.001)	7.602098∗ (0.000)	7.677452∗ (0.000)	37.770863∗ (0.000)

In the largest municipalities placed in the group 4, the population over 65 years and the population under 16 years are the determinant of public deficit in the short and in the long-run (see Table 10). Therefore, the social policies should be improved to reduce the pressure created by this segment of population.

**Table 10:** MMQ regression models to explain public deficit in Spanish municipalities from group 4 (2011–2020).

Variable in logarithm	Quantile levels				
	10%	25%	50%	75%	95%
GDP	2.107697 (0.669)	0.0714691 (0.846)	-0.0067978 (0.974)	-0.0640314 (0.609)	-0.1438643 (0.425)
Unemployment	2.038274 (0.242)	0.1137887 (0.383)	0.0398169 (0.589)	-0.0142759 (0.747)	-0.0897278 (0.162)
Participation	-0.443474 (0.951)	0.0053574 (0.992)	0.0226092 (0.941)	0.0352248 (0.847)	0.0528218 (0.841)
Herfindalh	0.8503768 (0.792)	0.0292732 (0.993)	-0.0022877 (0.987)	-0.025367 (0.757)	-0.0575594 (0.625)
Majority	1.199734 (0.624)	0.1596613 (0.381)	0.1196839 (0.247)	0.0904498 (0.145)	0.0496724 (0.578)
Party	-0.793547 (0.623)	-0.0034553 (0.977)	0.0269135 (0.693)	0.0491212 (0.231)	0.0800977 (0.175)
Pop15	-5.524063∗ (0.000)	-0.4229799∗ (0.000)	-0.2269087∗ (0.000)	-0.083529∗∗ (0.031)	-0.1164654∗∗ (0.049)
Pop65	-16.34306∗ (0.000)	-1.284172∗ (0.000)	-0.7053506∗ (0.000)	-0.2820802∗∗ (0.013)	-0.3083227∗∗∗ (0.076)
Constant	-26.79598 (0.513)	4.871821 (0.111)	6.089042∗ (0.000)	6.979151∗ (0.000)	8.220727∗ (0.000)

Robustness check is based on MG estimator and the results are consistent with MMQ regression models. Despite economic growth, public deficit continues to increase overall and in municipalities from group 1. Aspects related to political power like political participation, political party and Herfindalh index had the capacity to reduce deficit in overall sample (Table 11).

**Table 11:** MG estimators to explain public deficit in Spanish municipalities (2011–2020).

Variable in logarithm	overall sample	group 1	group 2	group 3	group 4
GDP	0.221355∗ (0.000)	0.227834∗ (0.033)	0.143738 (0.13)	0.125579 (0.14)	-0.053727 (0.703)
Unemployment	-0.00783327 (0.422)	0.006756 (0.436)	0.0127845 (0.78)	-0.042254 (0.109)	-0.021186 (0.707)
Participation	-0.977129∗ (0.000)	-0.403637∗ (0.000)	0.023382 (0.805)	-0.307257∗∗ (0.02)	0.038373 (0.853)
Herfindalh	-0.211298∗ (0.000)	-0.0788453∗ (0.000)	-0.094493 (0.15)	-0.05638 (0.335)	-0.033932 (0.667)
Majority	-0.015578 (0.322)	0.020389 (0.198)	0.020448 (0.7)	-0.001208 (0.982)	0.092722 (0.14)
Party	-0.021445∗∗ (0.03)	0.013637 (0.173)	-0.0157739 (0.503)	-0.033347 (0.179)	0.050034 (0.205)
Pop15	0.055639∗ (0.000)	0.0894528∗ (0.000)	-0.029472 (0.404)	0.027734 (0.382)	-0.0987233∗∗ (0.03)
Pop65	0.033287 (0.436)	-0.09112856∗ (0.005)	0.258744∗∗ (0.02)	-0.169934∗∗∗ (0.08)	-0.299367∗∗ (0.012)
Constant	7.622876 (0.000)	2.953448∗ (0.000)	5.883629∗ (0.000)	7.083476∗ (0.000)	6.564478∗ (0.000)

### 4.3 Causality analysis

The Juodis, Karavias and Sarafidis (2021) Granger non-causality test is applied on stationary data for overall sample and for each group of municipalities. The results in Table 12 for overall sample suggest causality from unemployment, political participation, Herfindalh index, majority, population less than 15 years and population more than 65 years. On the other hand, deficit growth is cause for economic growth, unemployment rate, political participation, majority, Herfindalh index, political party. The results at group level are different: in group 1, none of the variable is cause for deficit, but the deficit determines political participation and strength of governance. In group 2, political party, population less than 15 years and more than 65 years are causes for deficit growth. In group 2, there is a causal relationship from deficit to the following indicators: political participation, majority, political party. The results for group 3 indicate causal connection from growth, unemployment, political participation, majority of a party, population more than 65 years to deficit and from deficit to Herfindalh index and political party. In the case of group 4, economic growth and Herfindalh index are causes for deficit, while deficit is cause for growth, unemployment, political participation, majority and political part.

**Table 12:** The causalities between deficit and other variables in the analyzed municipalities (HPJ Wald statistics and associated p-values).

Causality	Overall sample	Group 1	Group 2	Group 3	Group 4
Δln(GDP per capita) → Δln(deficit)	1.3260 (0.2495)	0.0957 (0.7572)	1.4736 (0.2248)	8.4029∗ (0.0037)	4.43589∗∗ (0.0352)
Δln(Unemployment rate) → Δln(Deficit)	415.3547∗ (0.000)	0.1682 (0.6817)	0.7128 (0.3985)	67.8867∗ (0.000)	0.6387 (0.4242)
Δln(political participation)→ Δln(deficit)	243.1563∗ (0.000)	2.5314 (0.1116)	1.1766 (0.278)	4.6696∗∗ (0.0307)	2.1412 (0.1434)
Δln(Herfindalh index) → Δln(deficit)	85.3499∗ (0.000)	1.2473 (0.2641)	1.1001 (0.2941)	0.8466 (0.3575)	6.2460∗∗ (0.0124)
Majority→ Δln(deficit)	59.5189∗ (0.000)	0.4346 (0.5097)	0.1838 (0.6681)	21.3199∗ (0.000)	0.2117 (0.6454)
political party → Δln(deficit)	0.62704 (0.4284)	0.34422 (0.5574)	3.7399∗∗∗ (0.531)	0.1183 (0.7309)	0.0385 (0.8444)
Δln(population less than 15 years) → Δln(deficit)	220.3082∗ (0.000)	0.2723 (0.6018)	230.4577∗ (0.000)	1.5418 (0.2143)	1.6092 (0.2046)
Δln(population more than 65 years) → Δln(deficit)	14.5280∗ (0.0001)	0.1665 (0.6832)	3.1674∗∗∗ (0.0751)	2.9572∗∗∗ (0.0855)	0.3818 (0.5366)
Δln(deficit) →Δln(GDP per capita)	2118.7210∗ (0.000)	1.6434 (0.1999)	1.5322 (0.2158)	0.5057 (0.4770)	11.8865∗ (0.000)
Δln(deficit) → Δln(unemployment rate)	676.9874∗ (0.000)	0.4267 (0.5136)	1.7636 (0.1842)	1.2144 (0.2705)	17.4827∗ (0.000)
Δln(deficit) → Δln(political participation)	10.5655∗ (0.000)	3.2723∗∗∗ (0.075)	10.2314∗ (0.0014)	0.05390 (0.8164)	2.78093∗∗∗ (0.0954)
Δln(deficit) → Δln(Herfindalh index)	332.7655∗ (0.000)	3.8379∗∗∗ (0.0501)	0.5390 (0.4628)	12.3522∗ (0.0004)	1.610582 (0.2044)
Δln(deficit) → majority	227.118 ∗ (0.000)	1.3962 (0.2374)	286.122∗ (0.000)	0.89358044 (0.3445)	10.9945∗ (0.000)
Δln(deficit) → political party	198.564∗ (0.000)	0.4402 (0.5003)	156.334∗ (0.000)	5.9987∗∗ (0.0143)	56.3456∗ (0.000)

## 5 Conclusions and discussions

Spanish municipalities have had to control huge budget deficits in recent years, due to the difficulty of controlling different items of expenditure. They have also faced problems in obtaining funding. Spanish legislation obliges local authorities to be accountable and to maintain a balanced budget. However, the control of deficits and debt remains a complex problem despite the fact that budget stability, the origin of revenues and the sources of financing can be known through the analysis of the different budget items of revenue and expenditure. It is therefore necessary to understand the determinants of fiscal deficits, as persistent deficits quickly lead to the accumulation of public debt.

Among the determinants of deficits at the municipal level, political, economic and social factors have been considered. Since the size of municipalities might require different policies to manage the deficit problem, quantile regressions have been used for all municipalities. Separate models are also analysed by distinguishing four groups of municipalities according to the number of inhabitants.

It is found that economic growth only has a long-term beneficial effect on the deficit as it reduces the deficit at all quantile levels except at the 10% quantile. Unemployment, on the other hand, increases the deficit in both the short and long run.

The population over 65 and the political majority increase the deficit at the 75% and 95% quantiles. The population under 15 years of age increases the deficit in the short run by the 10% quantile, but in the long run it contributes to reducing the deficit. Children will in the long run become part of the labour force and become part of the labour market, thus contributing to deficit reduction by paying taxes. On the other hand, political participation and right-wing political parties contribute to the growth of the deficit in the higher quantiles.

The analysis suggests that in order to reduce the budget deficit, unemployment has to fall at the national level and economic growth has to be boosted. In addition, the quality of governance should improve and the policy agenda should aim at deficit reduction.

The results are different when analysing each group of municipalities separately. Thus, municipalities in group 3 (between 20,001 and 50,000 inhabitants) have to manage the problem of unemployment, while the dependent population under 16 years of age is the main difficulty for smaller and larger municipalities, generally in the short and long term. The population over 65 years of age is an important determinant of the deficit in group 2 and 4 municipalities.

Specific policies should target the main issue in each group of municipalities to reduce the deficit. Thus, municipalities in group 3 must manage the problem of unemployment, while in group 1 the economic level as measured by GDP must be improved. In the municipalities of groups 2 and 4, social policies must be improved in order to reduce the pressure created by the representative stratum of the population over 65 years of age.

The causality analysis allows us to formulate policy recommendations at national level and for each group of municipalities. At national level, unemployment should be controlled to alleviate the deficit issue, but political participation and strength, the existence of majority for a party play a significant role in reducing the deficit. In group 3, unemployment plays a significant role in deficit growth and active policies to reduce the number of unemployed persons should be a priority for this cluster of municipalities. There are large municipalities with 20,000–50,000 inhabitants that have the potential to attract foreign investment to support, for example, green sector and renewable energy sectors. The business environment should consider a flexible legislative background for investors to create new jobs. Political participation might play an important role by choosing a majority from a party that has a political program that supports the reduction of deficit. Political strength has the most significant impact on the reduction of deficit in the largest municipalities with more than 50,000 people.

The results of the study have important implications for managers in local governments and citizens. One of the most important of these is that political managers can control the deficit with different actions, for example by fighting unemployment or encouraging more economic activity. These actions, as our results show, reduce the deficit, and this will have a positive impact on citizens because their municipality will be able to spend more money on quality public services.

Councils provide public services that cannot always be measured in terms of cost, but in terms of public value and quality. There are services, such as those related to health, education or social services that do not fit with the principles of economy and efficiency. Therefore, managers in the public sector are faced with a choice: to provide a quality service, which implies higher spending, or to reduce the deficit and not spend. Political leaders must be able to deliver efficient, sustainable and rational services without ever losing quality. This can be achieved by focusing on those actions that allow deficit reduction without reducing spending.

Although these results shed light on the determinants of the deficit in Spanish municipalities, it would be useful to have more information on the management of municipalities to see if there are other relevant factors.

For future work, a more in-depth analysis could be carried out to determine whether the deficit is structural or cyclical. And the period before and after Covid-19 could be considered to find out if there has been any change in the determinants of the deficit due to the pandemic. In addition, we could study whether the consequences of the Ukraine-Russia conflict have an impact on the deficit.

## Declarations

### Author contribution statement

Javier Cifuentes-Faura: Conceived and designed the analysis; Analyzed and interpreted the data; Contributed analysis tools or data; Wrote the paper.

Mihaela Simionescu: Conceived and designed the analysis; Analyzed and interpreted the data; Contributed analysis tools or data; Wrote the paper.

Beata Gavurova: Conceived and designed the analysis; Analyzed and interpreted the data; Contributed analysis tools or data; Wrote the paper.

### Funding statement

Beata Gavurova was supported by Scientific Grant Agency of the Ministry of Education, Science, Research, and Sport of the Slovak Republic [Research project VEGA 1/0797/20].

### Data availability statement

Data will be made available on request.

### Declaration of interest’s statement

The authors declare no competing interests.

### Additional information

No additional information is available for this paper.

## Figures and Tables

**Table:** 

	GDP	Unemployment	Participation	Herfindalh	Majority	Political party	Pop15	Pop65	VIF
GDP	1								1.229
Unemployment	-0,0443	1							1.033
Participation	-0,0224	-0,0469	1						1.023
Herfindalh	0,0100	-0,0217	0,0290	1					1.225
Majority	-0,0193	-0,0155	0,0291	0,0712	1				1.296
Political party	-0,0502	-0,0885	0,0676	0,1354	0,1123	1			1.463
Pop15	-0,0655	0,0345	-0,0536	-0,0600	-0,0476	-0,0173	1		1.112
Pop65	-0,1292	0,0574	-0,0511	-0,0610	-0,0334	-0,1033	0,0986	1	1.334
